# Correlates of HCV seropositivity among familial contacts of HCV positive patients

**DOI:** 10.1186/1471-2458-6-237

**Published:** 2006-09-25

**Authors:** Giuseppe La Torre, Luca Miele, Alice Mannocci, Giacomina Chiaradia, Filippo Berloco, Maria L Gabrieli, Giovanni Gasbarrini, Maria Giovanna Ficarra, Antonio Matera, Gualtiero Ricciardi, Antonio Grieco, HCV-Southern Lazio Collaborative Group

**Affiliations:** 1 Institute of Hygiene, Catholic University of the Sacred Heart, School of Medicine "Agostino Gemelli", Rome, Italy; 2 Institute of Internal Medicine and Geriatrics, Catholic University of the Sacred Heart, School of Medicine "Agostino Gemelli", Rome, Italy; 3 Health Direction Policlinico "Agostino Gemelli", Rome, Italy

## Abstract

**Background:**

Determinants of intrafamilial HCV transmission are still being debated. The aim of this study is to investigate the correlates of HCV seropositivity among familial contacts of HCV positive patients in Italy.

**Methods:**

A cross-sectional study was conducted with 175 HCV positive patients (index cases), recruited from Policlinico Gemelli in Rome as well as other hospitals in Central Italy between 1995 and 2000 (40% female, mean age 57 ± 15.2 years), and 259 familial contacts. Differences in proportions of qualitative variables were tested with non-parametric tests (χ^2^, Yates correction, Fisher exact test), and a *p *value < 0.05 was considered significant. A multivariate analysis was conducted using logistic regression in order to verify which variables statistically have an influence on HCV positivity in contact individuals.

**Results:**

Seropositivity for HCV was found in 8.9% of the contacts.

From the univariate analysis, risk factors significantly associated to HCV positivity in the contacts were: intravenous drug addiction (p = 0.004) and intercourse with drug addicts (p = 0.005).

The only variables associated significantly and independently to HCV seropositivity in patients' contacts were intercourse with drug addicts (OR = 19.28; 95% CI: 2.01 – 184.94), the retirement status from work (OR = 3.76; 95% CI: 1.17 – 11.98), the time of the relationship (OR = 1.06; 95% CI: 1.00 – 1.11) and tattoos (OR = 7.68; 95% CI: 1.00 – 60.20).

**Conclusion:**

The present study confirms that having intercourse with a drug addict is the most significant risk factor for intrafamilial HCV transmission. The association with retirement status from work could be related to both a long-term relationship with an index case and past exposure to common risk factors.

## Background

Since its discovery in 1989, hepatitis C virus (HCV) has represented a major cause of chronic liver disease worldwide. The most recent WHO estimate of the prevalence of HCV infection is 2%, representing 123 million people. HCV is the leading cause of liver transplantation in developed countries, and the most common chronic bloodborne infection in the USA [1]. Although HCV is endemic in most parts of the world, there are significant geographic and temporal differences in the incidence and prevalence of HCV infection. Africa and Asia have the highest reported prevalence rates; while industrialized countries in North America, northern and western Europe and Australia have a lower prevalence. Nations with relatively low rates of HCV seroprevalence include Germany (0.6%), Canada (0.8%), France (1.1%), and Australia (1.1%). Low, but slightly higher seroprevalence rates have been reported in the USA (1.8%) and Japan (1.5–2.3%) [2]. The few studies on prevalence of HCV infection in Italy suggest an average value of 7–8%, increasing with age and from north to south [3-5]. With regards to developing countries, there is less data available about the burden of disease. Considering the most populous countries, China has a reported seroprevalence of 3.2%; India, 0.9%; while Egypt has the highest reported prevalence rate of 22% [2].

HCV infection appears in both acute and chronic clinical forms, but most of the morbidity is associated with chronic infection. 50–60% of HCV patients develop chronic hepatitis, which could result in severe liver disease, cirrhosis or even hepatocellular carcinoma [6]. However, assessing the incidence of HCV infection is difficult because most infections are initially asymptomatic (with only slightly increased ALT levels) [7].

The transmission of HCV infection usually involves the parental route. In fact, transmission through transfusions of blood or blood products from unscreened donors, injection drug use, unsafe therapeutic injections, occupational injury by needles or sharp instruments contaminated with blood, hemodialysis and tattooing is well documented [8,9]. However, at least 50% of all HCV positive patients do not have a history of blood transfusion or exposure to any other parenteral risk factor [10]. Therefore, it has been hypothesized that there must be other modes of HCV transmission in these patients. Kim et al. have previously found that horizontal transmission plays a major role in the spread of the hepatitis B virus (HBV) infection among family members of adults who are chronic carriers of HBsAg [11]. Familiar clustering of HCV infection has been demonstrated in epidemiological studies, but it is still controversial whether HCV can be transmitted through horizontal transmission to partners or other household contacts [10,12,13]. Furthermore, in Italy, despite increased frequency of HCV seropositivity in family members of patients with chronic HCV infection [14], the transmission of this virus through non-sexual household contacts has been regarded as uncommon [15]. As far as other vehicles of transmission is concerned, it is well known that HCV RNA titres in body fluids, such as saliva, semen, urine and vaginal fluids, are very low [16-18].

The aim of this study is to investigate the correlates of HCV seropositivity among familial contacts of HCV positive patients in Central Italy.

## Methods

### Patients and methods

A cross-sectional study was conducted with HCV positive patients (index cases), recruited from Policlinico Gemelli in Rome as well as other hospitals in Central Italy between 1995 and 2000, and their familiar contacts.

Sample size calculations (alpha = 0.05; beta = 0.20) suggested to sample size of at least 180 patients.

175 anti-HCV positive patients with chronic liver disease (identified as "index cases") were selected (97.2% of the eligible individuals) and a questionnaire was administered to them in order to evaluate their risk factors for HCV transmission. Then, the family members were tested for anti-HCV and interviewed to identify transmission risk factors. Participation was voluntary in accordance with the Guidelines for Observational Non-Interventional Studies from the Ethical Committee of the Gemelli Teaching Hospital. The research was conducted according to the Helsinki Declaration.

All 175 (70 females and 105 males, mean age 57 ± 15.2 years) patients who underwent a periodic health examination and tested positive for the antibody anti-HCV between July 1995 and December 2000 at the Policlinico "Gemelli", Ambulatory for Liver Disease, and at the other hospitals participating in the study in Southern Lazio (Central Italy) were enrolled in the study. Members of their family who were living together in the same house were studied. All patients had been living within this geographical area for at least ten years. All participants provided verbal consent to be in the study.

We enrolled 175 patients and 259 family members of 175 index cases into the study. Of these 175 patients, 142 (81.1%) had a clinical-histological diagnosis of chronic active hepatitis (CAH), 31 (17.7%) cirrhosis and 2 (1.1%) hepatocellular carcinoma (HCC). These patients were treated according to international therapeutical protocols.

All of the participants were assessed for HCV infection and liver disease by testing for anti-HCV antibody using a commercial ELISA, which was then confirmed by RIBA assay, alanine aminotransferase (ALT), aspartate aminotrasferase (AST), HBs Ag, HBs Ab, HBc Ab, polymerase chain reaction (PCR) as well as by ultrasound examination.

By means of a questionnaire, participants were asked about their past medical history, including surgical, dental and other possible risky procedures, blood or hemoderivates transfusions, hemodialysis, jaudice, tattoos, piercing and IV drug use.

### Statistical analysis

Quantitative variables were expressed as mean ± standard deviation and were compared by Student's t-test. Differences in the proportions of qualitative variables were tested with non-parametric tests (χ^2^, Yates correction, Fisher exact test), and a *p *value < 0.05 was considered significant. A multivariate analysis was conducted using logistic regression in order to verify which variables statistically had an influence on HCV positivity in contact individuals. The following variables were considered as covariates: gender [males (reference group) vs females], age group [<25 years; 25–49 years (reference group); ≥ 50 years]; job [manager; non-manual skilled; manual skilled (reference group); unemployed/student; retired from work]; intravenous drug users [yes vs no]; transfusions [yes vs no]; job with high risk of contamination [yes vs no]; surgical interventions with transfusions [yes vs no]; oral care [yes vs no]; hemodialitic treatments [yes vs no]; tattoos [yes vs no]; stable relationship with partner [yes vs no]; intercourse with drug addicts [yes vs no] and journey abroad [yes vs no].

We followed the procedure suggested by Hosmer and Lemeshow, using a stepwise approach (backward elimination). The goodness-of-fit of the model was assessed using the Hosmer-Lemeshow test for significance.

## Results

In table 1 the socio-characteristics of HCV patients and their contacts are shown.

The 259 household contacts (155 females, 104 males) had a mean age of 40.4 ± 19.0 years and included 99 heterosexual partners, 19 parents, 117 children, 10 siblings and 14 other relatives. Among these 23 were found to be positive for anti HCV. Eight of the anti HCV positive contacts had a known parenteral risk factor. The prevalence of anti HCV positivity in all household contacts was 8.9% (23/259). Excluding those with previous parenteral exposure, the proportion of anti HCV in family contacts was 6.0% (15/251). The highest prevalence was found in sexual partners (12.1%), followed by children (2.6%). The prevalence of anti-HCV increased with age (being highest in family contacts who were older than 60), as well in the sexual and non-sexual contacts groups. The proportion of anti HCV positive cases among household contacts with increased ALT and/or AST was 17.4% (4/23) versus 8.0% (19/236) among household contact with a normal ALT and/or AST (p = 0.132).

The prevalence of anti HCV in the contacts was not different when the clinical diagnosis of the index patients was taken into account. There was a significant difference in the duration of relationships with the index cases between anti-HCV positive contacts and anti-HCV negative contacts (35.2 ± 12 vs 26.5 ± 11.4). According to the distribution of HCV positive sexual contacts and to the duration of relationship, the detection frequency for anti-HCV increased in proportion to the duration of relationship.

It is interesting to note that the transmission of HCV was more likely in females (OR = 1.38; 95%CI: 0.52 – 3.74), however this pattern is not statistically significant.

In table 2 risk factors associated to HCV positivity in HCV patients and their contacts are shown. It can be deduced that the following variables are significantly associated to HCV seropositivity in patient contacts: intravenous drug use (p = 0.004) and intercourse with drug addicts (p = 0.005). As far as the differences in HCV seropositivities between index cases and their contacts is concerned, there was no significant different pattern in risk factors, with only blood transfusions approaching significance (26% in HCV patients vs. 4.8% in their contacts; p = 0.06).

The prevalence of anti-HCV among household contacts, excluding cases with previous parenteral exposure, in this study was 6.2%. These results are higher than in other studies but are similar to the prevalence of anti-HCV in our control group (5.2%). These results support the hypotesis that Southern Lazio is an area with an elevated distribution of HCV. Moreover, the prevalence of anti-HCV in sexual contacts (12.1%) was higher than in non sexual contacts (2.5%), but not so high as to suggest that HCV can be sexually transmitted.

The higher prevalence among older contacts may be due to the duration of their relationship with the index case but also to the re-use of medical materials (syringes for intramuscle injections) administered at home (once common in Italy).

The substantial percentage of seropositive contacts with no identifiable risk factors supports the hypothesis of unapparent parenteral transmission of the virus.

In table 3 the results of the multivariate analysis are presented. After controlling for age and gender, the only variables associated significantly and independently to HCV seropositivity in patients' contacts are intercourse with drug addicts (OR = 19.28; 95% CI: 2.01 – 184.94), retirement status (OR = 3.76; 95% CI: 1.17 – 11.98), the length of time of the relationship (OR = 1.06; 95% CI: 1.00 – 1.11) and tattoos (OR = 7.68; 95% CI: 1.00 – 60.20). After the exclusion from the analysis of those contacts with known parenteral exposure we found similar trends in the risks (table 4), with an increment of risk observed for people that had tattoos (OR = 29.09).

It's interesting to remark that a stable sexual relationship with a HCV positive patient did not affect the seropositivity in the contacts (OR = 0.86; 95% CI: 0.13 – 5.49).

## Discussion

Accurate epidemiological data about the diffusion and transmission of HCV infection are necessary in order to understand the burden of the disease. Assessment of HCV infection prevalence and the identification of all of the associated risk factors are required in order to plan strategies that will interrupt the different patterns of HCV transmission. It is widely documented that the parental route is the most important route of HCV transmission: injection drug use, blood transfusions and other unsafe health-care related procedures are the most frequently mentioned risk factors. Injection drug use is generally considered to be the major risk factor of new infections in developed countries, while transfusions and unsafe therapeutic injections still represent the main source of infection transmission in developing areas [19].

On the other hand, data concerning sexual and intrafamilial transmission remain unclear and the role played by the family in the spread of HCV infection has yet to be defined. Some case reports have been published that clearly demonstrate sexual transmission of HCV [20,21]. In addition some epidemiological and case-control studies have confirmed that intrafamilial transmission occurs [3,22,23], while others have argued that household exposure non-significantly increases the risk of HCV positivity [24-26]. This difference in the results of different studies may be a consequence of several factors such as the ELISA tests used to detect anti-HCV antibodies (first and second-generation ELISA used in previous studies have a relatively low sensitivity in relation to the newer third-generation assays), geographic area, viremia levels, sexual behaviors of the subjects studied, selection of the studied population, statistical methods used to assess within-household clusters of HCV infection and genotypes.

We conducted a cross-sectional study on the intrafamilial transmission of HCV. In our study the prevalence of anti HCV positivity in all household contacts was 8.9%, which is higher than in the general population, which is in agreement with other authors. The highest prevalence was found in sexual partners (12.1%), followed by children (2.6%). Excluding those with previous parenteral exposure, the proportion of anti-HCV antibodies in family contacts was 6.2%. These results are similar to the prevalence of anti-HCV in our control group (5.2%), supporting the hypothesis that Southern Lazio is an area with an elevated level of HCV in circulation [27]. Moreover, the prevalence of anti-HCV in sexual contacts (12.1%) was higher than in non sexual contacts (2.5%), suggesting that HCV could be sexually transmitted. But re-analyzing data removing those contacts with known parenteral exposure, we found that stable sexual relationships cannot be considered a risk factor. Interestingly, the duration of the relationship plays a fundamental role in all of the multivariate analysis models, suggesting that the difference in the prevalence rates among sexual and non sexual contacts could be explained simply by a time factor. Beside this, we found interestingly that being retired from work is an independent factor associated to HCV positivity among contacts of HCV chronic patients. This result does not concur with other studies that have shown that sexual transmission of HCV may occur in spouses, while it does confirm that the spreading of the virus to other family members seems to be rare [28]. However, there are conflicting data in the scientific literature regarding sexual diffusion of HCV. Evidence that supports the hypothesis for this mode of transmission is that in genotyping the HCV, some authors have detected the same genotype in the spousal couple [23,24,29,30]. This is in contrast with the results obtained from other studies in which the same genotype was found in very few couples, suggesting that the risk of sexual transmission was low [31]. Moreover, the finding that many of the cases of intrafamilial spread involved siblings suggests that shared use of domestic objects may played a more important role than sexual transmission [32]. However, this method of transmission does not appear to be predominant when compared with the transmission of other viruses (HIV and HBV) which can be transmitted by both vertical and horizontal (sexual and non-sexual) means [33].

Furthermore, we investigated the duration of the relationship with the index cases between anti-HCV positive contacts and anti-HCV negative contacts. According to the distribution of HCV positive sexual contacts and the duration of the relationship, the frequency of detection for anti-HCV increased in proportionately with the duration of the relationship. This result agrees with the finding of other studies that anti-HCV positivity increases with the length of the marriage [29,30,34,35].

The higher prevalence among older contacts may be due to the duration of the relationship with index cases but also to some behaviors once common in Italy, such as the re-use of medical material (syringes for intramuscle injections) administered at home [36].

Among the risk factors that we analyzed with multivariate analysis, those significantly associated with HCV positivity in household contacts were being a drug addict and having intercourse with drug addicts. Moreover, the length of exposure and the status of the partner could also explain the fact that the retirement status is associated with HCV positivity. As far as drug addiction is concerned, our survey confirms the findings of other Italian studies [15,28,37-39].

In a recent review, Ackerman and coll. [40] found that intrafamilial HCV transmission is associated with the severity of liver disease in the index patients, the number of family members infected with HCV, the duration of exposure and sexual contact with the index patient. Interestingly, out of 67 studies selected in this review, 25.4% were from Italy. This cross-sectional study confirms that tattoos are a means of HCV transmission [8].

This study has several limitations. First of all, it is a cross-sectional study and it was not possible to assess the time-relationship between types of exposure and outcome (HCV seropositivity), as in a cohort study. As an example, we are aware that independent infection among family members cannot be excluded, and temporal trends cannot be determined. Anyway, we do know that index cases are long-term patients so it is likely that family members became HCV positive after the cases did.

As far as information bias is concerned, this type of error could have been avoided as we used a very well established means of collecting information on past exposures and risk factors. With regards to selection bias, we collected information regarding the contacts of the index patients attending our Hepatology Ambulatory in Rome and from other hospitals in Southern Lazio, and we believe that our population is a random sample of patients with HCV in this Region.

Another limitation of the study refers to the lack of assessment for HCV genotype, so we were not able to address whether differing genotypes have differing rates of transmission.

In conclusion, the substantial percentage of seropositive contacts with no identifiable risk factors support the hypothesis of unapparent parenteral transmission of the virus.

Furthermore, the results of such analyses are useful in generating hypotheses about possible causes of disease, rather than drawing defined conclusions [41].

## Conclusion

Identification of risk factors for HCV infection and strategies based on these findings have helped contain HCV infection in many developed countries. However, results from data on HCV spread through sexual or non-sexual means in household contacts still remains to be fully explained.

## Competing interests

The author(s) declare that they have no competing interests.

## Authors' contributions

All of the authors participated in the establishment of the research. AG conceived the study, participated in its design and coordination. GLT and AM performed the statistical analysis. GLT, LM and AM wrote the initial draft of the manuscript, which GLT, GC and AG edited. All authors read and approved the final manuscript.

## Pre-publication history

The pre-publication history for this paper can be accessed here:



## Figures and Tables

**Table 1:** Socio-demographic characteristics of Patients HCV positive and their Contacts.

**Variables**	**HCV patients**	**Contacts**		p *
			
		**HCV positive**	**HCV negative**	
**Age **(standard deviation)	55,77 (15,197)	50,52 (18,81)	39,13 (19,05)	0,07
**Gender**				
Males	105 (60.0%)	8 (34,8%)	93 (42,5%)	0,447
Females	70 (40,0%)	15 (65,2%)	126 (57,5%)	
**Province of residence**				
Caserta	15 (8,6%)			
Frosinone	43 (24,6%)			
Latina	117 (65,4%)			
**Occupational Activity**				
Manager	5 (2,9%)	1 (4,3%)	17 (7,8%)	0,072
Non Manual skilled	31 (17,7%)	1 (4,3%)	35 (16,1%)	
Manual skilled	73 (41,7%)	10 (43,5%)	79 (36,2%)	
Unemployed/Student	12 (6,9%)	4 (17,4%)	61 (28,0%)	
Retired from work	54 (30,9%)	7 (30,4%)	26 (11,9%)	

* p value refers to tests of significance within the contacts' group

**Table 2:** Risk factors associated to HCV seropositivity

**Variables**	**HCV patients**	**Contacts**		p *
			
		**HCV positive**	**HCV negative**	
**Intravenous drug addiction**				
Yes	14 (9,3%)	2 (9,5%)	2 (0,9%)	0,004
No	136 (90,7%)	19 (90,5%)	209 (99,1%)	
**Blood transfusions**				
Yes	39 (26,0%)	1 (4,8%)	10 (4,8%)	0,996
No	111 (74,0%)	20 (95,2%)	199 (95,2%)	
**Job at risk of contamination**				
Yes	7 (4,7%)	1 (4,8%)	8 (3,8%)	0,837
No	142 (95,3%)	20 (95,2%)	200 (96,2%)	
**Surgical intervention with blood transfusions**				
Yes	28 (19,0%)	1 (4,8%)	8 (3,8%)	0,826
No	119 (81,0%)	20 (95,2%)	203 (96,2%)	
**Dental care**				
Yes	112 (75,7%)	15 (71,4%)	154 (74,4%)	0,768
No	36 (24,3%)	6 (28,6%)	53 (25,6%)	
**Hemodialytic treatments**				
Yes	0	0	1 (0,5%)	0,749
No	148 (100%)	21 (100%)	205 (99,5%)	
**Tattoos**				
Yes	6 (4,1%)	2 (9,5%)	6 (2,9%)	0,111
No	142 (95,9%)	19 (90,5%)	204 (97,1%)	
**Stable relationship with partner**				
Yes	123 (83,1%)	17 (81,0%)	142 (67,9%)	0,219
No	25 (16,9%)	4 (19,0%)	67 (32,1%)	
**Intercourse with drug addicts**				
Yes	5 (3,4%)	2 (9,5%)	2 (1,0%)	0,005
No	141 (96,6%)	19(90,5%)	198 (99,0%)	
**Journey abroad**				
Yes	62 (41,3%)	8 (38,1%)	74 (35,6%)	0,819
No	88 (58,7%)	13 (61,9%)	134 (64,4%)	

* p value refers to tests of significance within the contacts' group

**Table 3:** Results of the logistic regression analysis.

**Variables in the model**	**OR°**	**95% CI**	**p**
*Drug addiction*			
No (reference group)	1		
Yes	19.28	2,01 – 184,94	0,010
*Job*			
Manual skilled (reference group)	1		
Retired from work	3.76	1.18 – 11.99	0.025
*Time of relationship*	1.06	1.01 – 1.11	0.038
*Tattoos*			
No (reference group)	1		
Yes	7.68	1.00 – 60.20	0.05

(dependent variable: HCV seropositivity in patients' contacts).

°Adjusted for age and sex

Hosmer-Lemeshow Test: p = 0.459

**Table 4:** Results of the logistic regression analysis, without patients with known parenteral exposure.

**Variables in the model**	**OR°**	**95% CI**	**p**
*Job*			
Manual skilled (reference group)	1		
Retired from work	3.61	1.11 – 11.78	0.033
*Time of relationship*	1.07	1.01 – 1.13	0.018
*Tattoos*			
No (reference group)	1		
Yes	29.09	3.28 – 257.99	0.002

(dependent variable: HCV seropositivity in patients' contacts)

°Adjusted for age and sex

Hosmer-Lemeshow Test: p = 0.215
